# A Case of Success With Immunotherapy After Changing the Therapeutics Strategy in Non-small Cell Lung Cancer

**DOI:** 10.7759/cureus.47874

**Published:** 2023-10-28

**Authors:** Bárbara Machado, Inês Soares de Pinho, Ana Rita Aranha, Viktor Malyarchuck, Joana Godinho

**Affiliations:** 1 Medical Oncology, Centro Hospitalar de Entre Douro e Vouga, Santa Maria da Feira, PRT; 2 Medical Oncology, Centro Hospitalar Universitário Lisboa Norte, Lisbon, PRT

**Keywords:** survival benefit, definitive radiotherapy, pembrolizumab, immunotherapy, metastatic non-small cell lung cancer

## Abstract

Immune checkpoint inhibitors (ICI) have already shown benefit with higher response and survival rates when compared to standard chemotherapy in advanced non-small cell lung cancer (NSCLC). Although there is evidence that radiation and immunotherapy offer good response rates without additional toxicity, these treatments are not currently utilized in our everyday clinical practice to treat advanced disease. We present a case of success of a 50-year-old male with stage IIIC adenocarcinoma of the lung with high PD-L1 expression and no driver mutations whose disease progressed after two cycles of induction chemotherapy. After that, he started systemic treatment with pembrolizumab monotherapy, and there was such a good response that he proposed definitive radiotherapy for the only remaining pulmonary lesion. Stereotactic body radiation therapy (SBRT) was performed with no major toxicity. He is alive, in follow-up for more than two years, with no signs of active oncological disease. Our case represents an example of success, demonstrating a great tumor response with immunotherapy that allowed a patient with advanced non-metastatic NSCLC whose disease had progressed with platinum-based chemotherapy to get radical treatment with SBRT. The failure of the first-line treatment can result in more investigation on the efficacy and benefits of beginning treatment of these kinds of tumors with ICI directly.

## Introduction

Lung cancer is the second most common cancer worldwide, with 2.21 million new diagnoses in 2020, and the leading cause of cancer deaths with 1.80 million deaths in 2020 [1]. Non-small cell lung cancer (NSCLC) represents the majority of cases [2], with adenocarcinoma being the most frequent subtype [3]. NSCLC is frequently detected at an advanced stage of the disease [3]. A third of patients are found to have a locally advanced NSCLC [4,5], which includes a broad spectrum of diverse clinical features [4]. The standard of care for patients with an unresectable stage III NSCLC and a good Eastern Cooperative Oncology Group performance status (ECOG PS) is platinum-based doublet chemotherapy with concurrent radiation therapy (chemoradiotherapy) [4,5]. Even in cases where chemoradiotherapy proves to be successful, the median progression-free survival (PFS) is low, and only 15% of the patients remain alive after five years [5]. If the patient is not able to receive the multimodality treatment, radiation therapy alone results in an estimated 5% five-year overall survival (OS), which is still a very poor outcome [4]. This survival rate is equivalent to the 5% five-year survival rate estimated for stage IV disease [3]. On the other hand, in most stage IV cases, systemic treatment is chosen for patients with an ECOG PS of 0-2 [6]. The NSCLC systemic treatment in advanced disease (inoperable stage III and stage IV disease) has changed significantly in recent years, due to the discovery of immune checkpoint inhibitors (ICIs) [7]. ICI showed improvement in the OS and PFS of patients and an improvement in their quality of life too (compared to chemotherapy) [7]. Therefore, consolidation immunotherapy with durvalumab is recommended for patients with stage III unresectable cancer who received concurrent platinum-based chemoradiotherapy without disease progression and whose tumor proportion score (TPS) PD-L1 is ≥ 1 [7]. On the other hand, systemic treatment is selected as well based on PD-L1 status in patients with stage IV disease who do not display any specific tumor-driver mutation [6]. In the absence of contraindications, the first-line treatment for patients with ECOG PS 0-1 without oncogenic driver mutations usually consists of a combination of platinum-based chemotherapy plus a programmed cell death receptor 1 (PD-1)/PD-L1 blockade. In patients with high PD-L1 expression (TPS ≥ 50% or for atezolizumab, combined proportion score (CPS) ≥ 10%), an ICI-chemotherapy combination or a single-agent ICI may be used. It is uncertain whether to prioritize ICI-chemotherapy combinations or rather favor PD-(L)1 blockade alone, so both treatments are valid options [6].

Immunotherapy 

ICIs are a type of immunotherapy. They are monoclonal antibodies that interrupt the inhibition of immune signaling pathways, by allowing the immune cells, especially cytotoxic T lymphocytes, to recognize tumoral neoantigens and eliminate malignant tumor cells [7-9]. They block immune checkpoints, by targeting T-cell receptors or their ligands, such as cytotoxic T lymphocyte-associated molecule-4 (CTLA-4), PD-1, and PD-L1 [8,9]. Within the lymph node, CTL-4 is a coinhibitory molecule expressed in T-cells that suppresses cytotoxic T lymphocytes and boosts regulatory T-cell activity. On the other hand, within the tumor microenvironment, PD-1, after binding with PD-L1, regulates immune tolerance by suppressing T-cell receptor-mediated lymphocyte proliferation and cytokine secretion [2,8,9], contributing to the prevention of autoimmune events [9]. There are three major ICI classes approved: anti-PD-1, including nivolumab and pembrolizumab; anti-PD-L1, such as durvalumab or atezolizumab; and anti-CTL-4, such as Ipilimumab, among others [7,9]. Regarding locally advanced NSCLC, the purpose of the PACIFIC study was to evaluate durvalumab or placebo in patients with unresectable stage III non-small cell lung cancer that did not progress after concurrent chemotherapy [5,7]. With a 36-month disease-free survival (DFS) of 17.2 versus 5.6 months (placebo) and a greater response rate (30% vs. 17.8%, respectively), this trial demonstrated the advantage of durvalumab. The OS was similarly better, with a 36-month OS of 57% as opposed to 43.5% (placebo) [7]. However, despite the DFS improvement in this subgroup, no OS impact was confirmed in the PD-L1 negative subgroup (<1%) when an ad hoc analysis was conducted. Following these findings, the FDA approved durvalumab regardless of PD-L1 expression; however, the EMA only approved it in this context for patients with TPS PD-L1 expression ≥ 1% [7]. In advanced disease, the KEYNOTE-024 study was the first study to prove the benefit of pembrolizumab in patients whose tumors presented PD-L1 (TPS) expression higher than 50% and no epidermal growth factor receptor (EGFR) or anaplastic lymphoma kinase (ALK) mutations. It was compared with standard chemotherapy with an increase in survival. After the KEYNOTE-042 study failed to demonstrate statistical significance in PFS with single-agent pembrolizumab in patients with PD-L1 expression between 1% and 49%, monotherapy pembrolizumab was approved as a first-line palliative treatment option for patients with NSCLC and PDL-1 TPS of more than 50% in tumor cells (both FDA and EMA approval in this setting) [7]. Therefore, pembrolizumab is a treatment option in patients with advanced NSCLC and high PD-L1 expression [6]. Additionally, ICIs are well-tolerated and have specific immune-related toxicities that can affect any organ, at any time and most are usually manageable. There are some severe toxicities that require more complex approaches, such as myocarditis and neurological syndromes, but they are rare [8]. One of the most common toxicities is endocrinopathies [9]. It is important to remember that, despite the positive results, only a minority of patients derived a very long-term benefit from ICI treatment [10].

Radiotherapy

The effects of radiation on cancer's immune cycle are already known. The cells killed by radiation release tumor-associated antigens and cytokines that attract T cells and lead to improved priming and activation of effector T cells [11,12]. Additionally, there is an increased expression of surface molecules on the irradiated cells that makes them more vulnerable to cytotoxic T-cell-mediated cell killing [11,12]. These mechanisms are responsible for the recognition and elimination of tumor cells by antitumor lymphocytes, even in distant metastasis [13]. Some cases of systemic effect on metastasis after local intervention with radiotherapy (RT) have been described, which is called the abscopal effect [2,13].

RT has always been regarded as a highly effective but local therapy for cancer [11]. Stereotactic body radiation therapy (SBRT) is revolutionary as a non-invasive curative therapy that has become the standard therapy for medically inoperable located stage I and II NSCLC [11]. It improves survival with local control superior to 90% and has minimal toxicity [2,11]. Intensive regimes with a biologically effective dose equivalent or superior to 100 Gy have better local control and survival (versus less intensive regimens). In general, three to five fractions of SBRT are used to treat lung cancer. However, additional fractions may be required in certain cases, such as extremely central tumors [14].

Combination of immunotherapy and radiotherapy 

The safety and efficacy of combining radiotherapy and immunotherapy in NSCLC were verified in several clinical trials [15]. Pre-clinical studies point to RT as a primary event for immunotherapy and a synergistic activity between radiation and PD-1 inhibition [2,15]. The beneficial effect may be explained by the effects of radiotherapy in the tumor microenvironment, by inducing cell death and releasing antigens that will activate T cells [2,15]. Some advanced solid tumors are known to have a T-cell-inflamed tumor microenvironment called tumor-infiltrating lymphocytes (TILs), such as NSCLC. Tumors with a higher proportion of TILs are called “hot tumors” and are associated with favorable response to immunotherapy and with a better prognosis [15]. When these antigens are released and T cells are recruited, the tumor may switch from “cold” to “hot”, being more likely to respond to immunotherapy [15].

Moreover, RT may overcome resistance to ICIs, and the abscopal effect has been described with RT and immunotherapy [2]. The main precautions with this combination are the overlapping toxicities, especially pneumonitis, in the case of thoracic radiotherapy. However, the combination does not appear to produce significantly increased toxicities beyond those associated with each treatment independently [2].

## Case presentation

**Figure 1:**
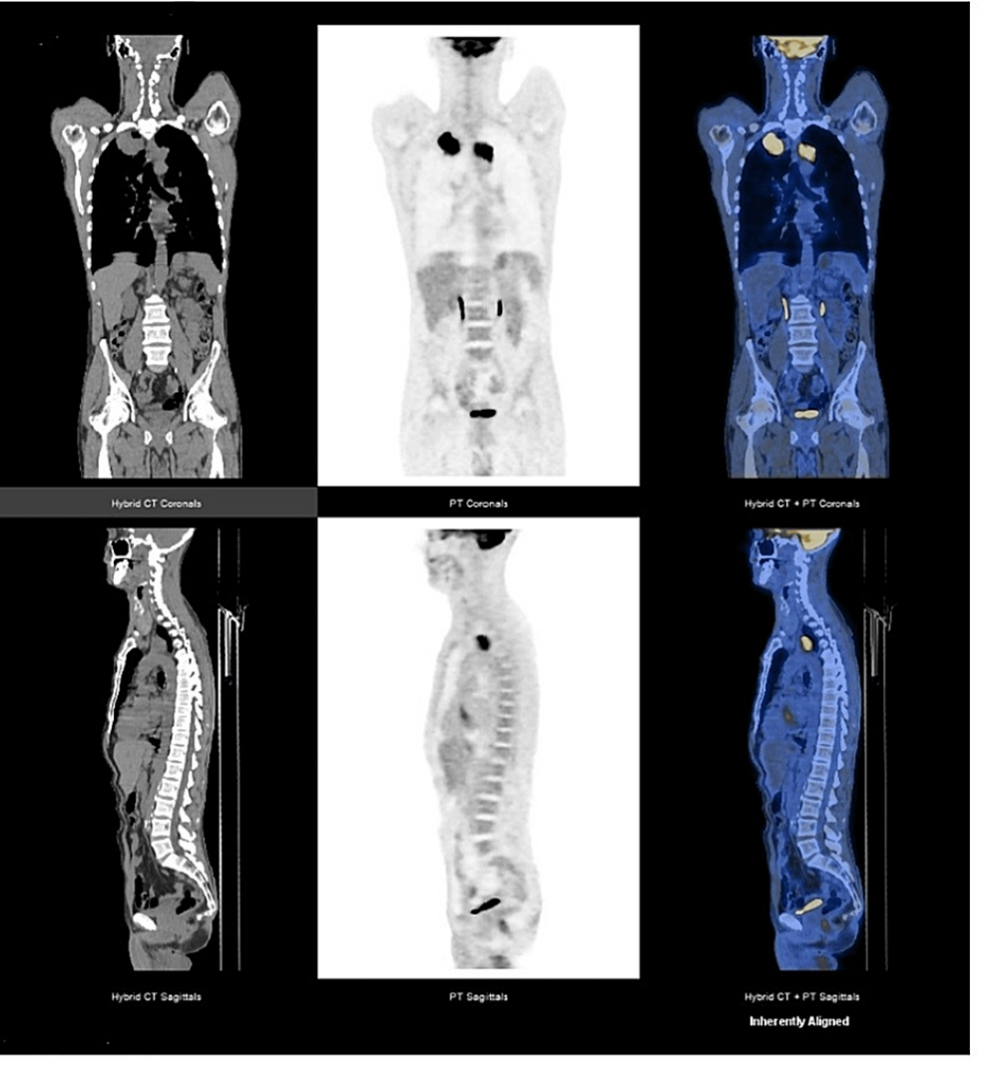
Staging FDG/PET-CT performed in May 2020

We present the case of a Caucasian, 50-year-old male, fit (ECOG PS 1), heavy smoker (70 pack-year) with no relevant past medical history, nor regular medication. He was observed in the Emergency Department with pleuritic chest pain, cough, hemoptoic sputum, asthenia, and night sweats that had been evolving for several days. He was also suffering from left lower limb claudication, pain, and swelling. The patient was hemodynamically stable, with a normal oxygen saturation level. Physical examination showed edema, redness, and pain in the left lower limb. Laboratory tests revealed anemia (hemoglobin level of 12.4 g/dL), leukocytosis (leucocyte count of 12,400/uL), and high levels of C reactive protein (115 mg/L) and of D-dimer (12,333 ng/mL). A Doppler ultrasound was performed and revealed thrombosis in multiple deep veins in the middle and distal thirds of the leg. A chest computed tomography (CT), ordered to exclude a pulmonary embolism, showed a pulmonary mass (72 x 47 mm) in the right upper lobe, bilateral mediastinal and hilar adenopathies, and a mediastinal mass involving the left subclavian artery. The patient was hospitalized in the Service of Pneumonology, due to the suspicion of a thoracic tumor and cancer-associated thrombosis. During the hospitalization, he was started on enoxaparin, which continued after medical discharge, with posterior resolution of the thrombosis. During hospitalization, the 18-fluoro-D-glucose-PET-CT (FDG/PET-CT) showed intense FDG uptake in the right upper lobe mass (SUVmax: 30.7) and in an upper-left paraoesophageal paratracheal mass (SUVmax: 19) (Figure 1). The transthoracic biopsy made the diagnosis of a lepidic growth invasive adenocarcinoma. A molecular cancer study reported a PD-L1 expression (TPS) between 80% and 90% and no target tumor-driver mutations. An endoscopic bronchial ultrasound (EBUS) was requested and confirmed that the left paratracheal mass was an adenopathy. Therefore, the final diagnosis was a stage IIIC lung adenocarcinoma.

**Figure 2:**
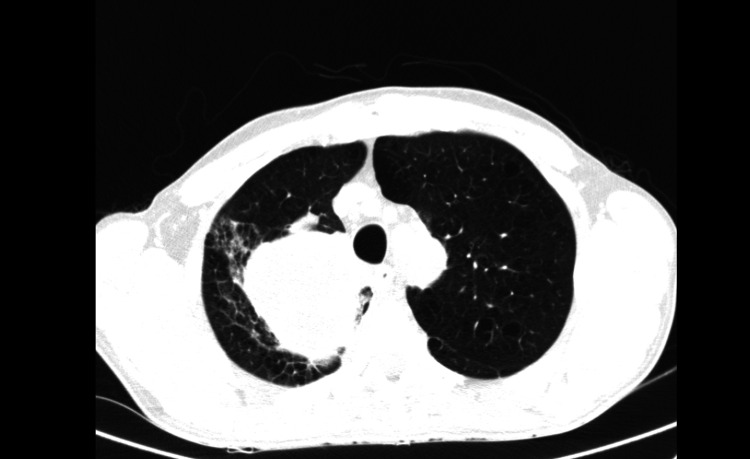
Follow-up abdominal and chest CT after two cycles of chemotherapy in August 2020

**Figure 3:**
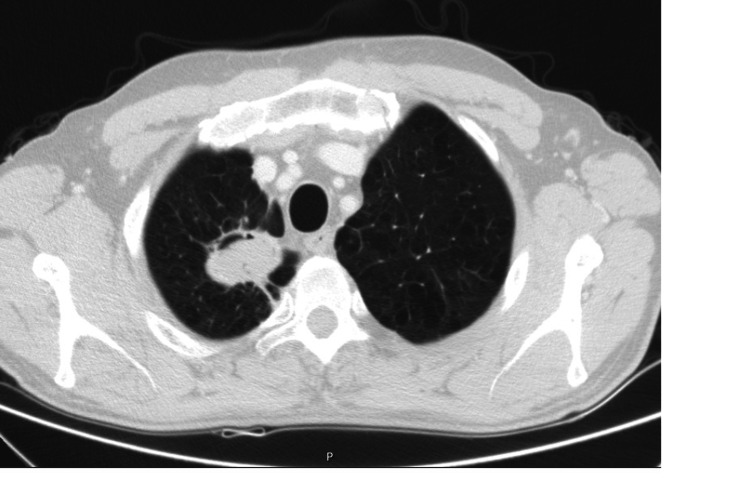
Follow-up abdominal and chest CT after 15 cycles of pembrolizumab in July 2021

**Figure 4:**
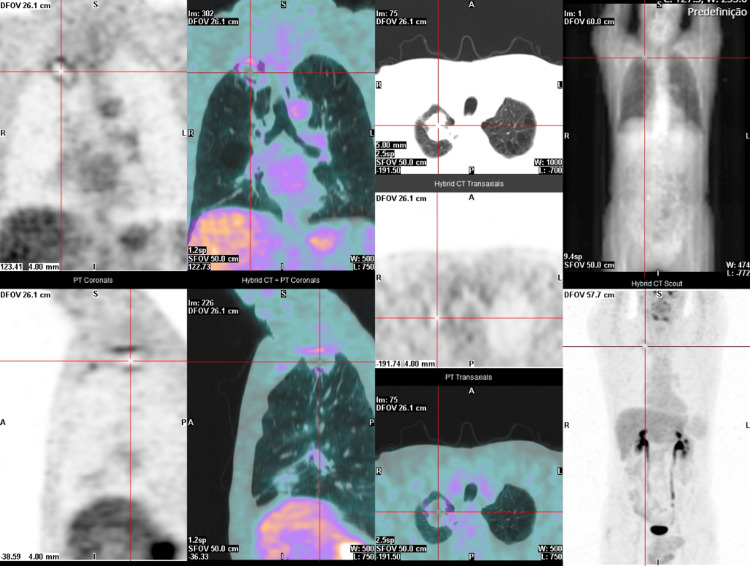
FDG/PET-CT after 16 cycles of pembrolizumab in August 2021

**Figure 5:**
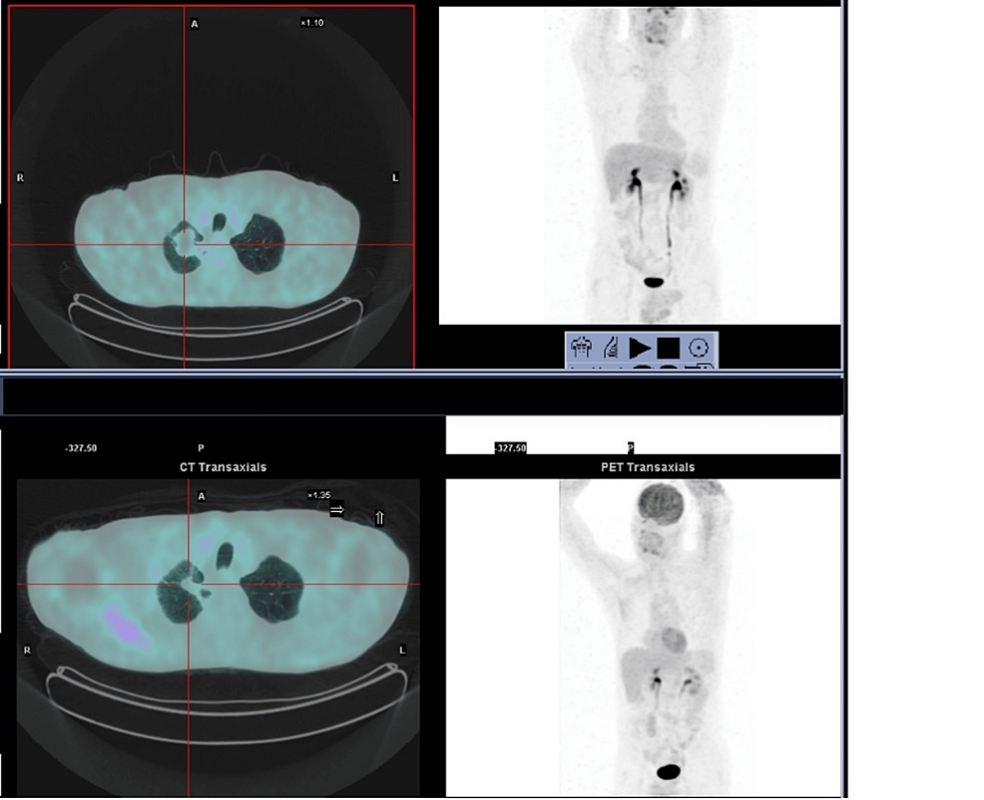
FDG/PET-CT after pembrolizumab and SBRT to the lung mass in April 2022

**Figure 6:**
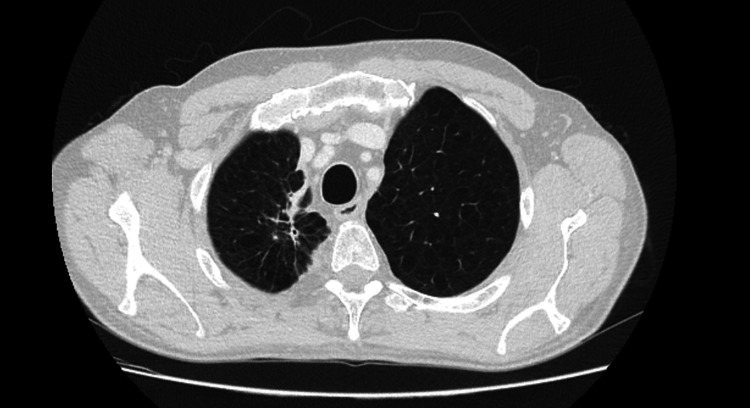
Follow-up abdominal and chest CT after finishing pembrolizumab and receiving SBRT performed in December 2022

He was observed at a medical oncology consultation, presenting with an ECOG PS 1, dyspnea, pleuritic chest pain, cough, hemoptoic sputum, asthenia, loss of weight, and fatigue. Our lung cancer tumor board decided to propose the patient for sequential chemoradiotherapy with a curative intent. He started cisplatin and paclitaxel in July 2020. However, after two cycles, an abdominal and chest CT was performed and revealed disease progression with a marked dimensional increase of the tumor (measuring 8.7 x 8.4 x 8.5 cm), the persistence of the left paramediastinal mass (3.2 x 2.9 cm), and of the bilateral mediastinal and hilar adenopathies (Figure 2). The case was examined in the lung tumor board, and palliative treatment was decided on due to the disease's progression throughout chemotherapy. As a result, the primary therapeutic objective was modified, and a systematic palliative care strategy was put forward. He began pembrolizumab in November 2020. In July 2021, after 15 cycles, follow-up abdominal and chest CT showed a partial response with right upper lobe lung mass reduction (40 x 37 mm) and no mediastinal or hilar adenopathies (Figure 3). An FDG/PET-CT was done and revealed FDG uptake only in the right upper lobe lung mass (SUVmax: 2.7) (Figure 4). Following these results, the multidisciplinary tumor board decided to perform a definitive local therapy with SBRT on the primary tumor. The patient received a total dose of 50 Gy divided in five fractions to the upper right lobe between the 13th and 22nd of October 2021, with no immediate toxicity. An FDG/CT-PET performed after the RT treatment (in April 2022) displayed densification in the right superior lobe (where previously there was a mass or lesion) with morphometabolic improvement (SUVmax: 1.6) (Figure 5). He continued pembrolizumab until the 35th cycle (October 2022). Treatment was well-tolerated during the two years in spite of having recorded some immune-related toxicities: hyperthyroidism G2 managed with propranolol and metibasol (he was referred to an endocrinology consultation for additional study), primary adrenal insufficiency G2 managed with 5 mg per day prednisolone, and diarrhea G2 resolved with low diet fiber, loperamide, and an increased dose to 10 mg prednisolone per day. Follow-up abdominal and chest CT was performed in December 2022 and revealed an irregular densification at the apex of the right lung, globally overlapping with previous exams (Figure 6). In the last medical observation in July 2023, the patient had an ECOG PS 0, did not have respiratory symptoms, had gained some weight, had started to work again, and had an active life. Another follow-up CT was made in July 2023, not showing any signs of oncologic disease. Thus far, after 35 months from the beginning of immunotherapy and 24 months after radiotherapy treatment, there has been no evidence of progression or active cancer disease.

## Discussion

There are several points to highlight in this case. First, we must underline the treatment's success. Our patient had an advanced NSCLC that progressed while receiving chemotherapy, impeding the completion of the required chemoradiotherapy to cure the disease. Thus, the prognosis was poor. Following this, the goal of the treatment was shifted to palliative care, and pembrolizumab was chosen as the initial course of treatment in this context due to the patient's high PD-L1 expression, good ECOG PS, and accessibility to therapy in Portugal. Treatment results exceeded our expectations. The patient achieved a partial response in 8.5 months of treatment with lung mass regression and disappearance of all other masses, results that are not usually seen with systemic treatment. With SBRT, we were able to radicalize the remaining primary lesion due to this exceptional response to pembrolizumab. This was accomplished with good treatment tolerance.

Secondly, after this treatment combination, our patient is still alive with no disease progression for 35 months after starting treatment with pembrolizumab. In the KEYNOTE-042 trial, which included locally advanced and metastatic NSCLC, pembrolizumab had a major benefit in the PD-L1 (TPS) ≥ 50% population, with a median OS of 20 months and a median PFS of 6.5 months after five-year follow-up (vs. chemotherapy) [16]. Thus, our patient not only had a great response to pembrolizumab, but its survival was enhanced due to the local treatment with radiotherapy for the remaining metabolically active lesion. In addition, our patient is still responding (durable response observed in the KEYNOTE 042 trial was 28.1 months in the pembrolizumab group with PD-L1 (TPS) ≥ 50%) [16] and was able to complete the 35 cycles of treatment. Nevertheless, the definition of durable response remains controversial and so does the optimal duration of immunotherapy [17]. It was questionable whether to continue immunotherapy in this patient after receiving radiotherapy. Based on available studies, patients with advanced non-small lung cancer receiving first-line immunotherapy should continue immunotherapy for two years [17]. We, therefore, made the decision to continue therapy until the 35 cycles were completed because there was insufficient evidence to support stopping it and no serious toxicity occurred. The expected benefits of combining immunotherapy and RT were also another reason to maintain immunotherapy to continue to potentiate the effects of RT. However, more prospective studies are required to inform us of the benefits of continuing immunotherapy in this setting. As shown before, testing for PD-L1 status is the standard of care in NSCLC, and these trials proved substantial evidence from correlating levels of PD-L1 expression with response and clinical efficacy [18]. However, PD-L1 remains a controversial biomarker of immunotherapy response and several issues limit its utility [18].

Another major point to discuss is that we have not found case reports of radiotherapy being used to radicalize treatment after a good response to immunotherapy. Most trials include RT before or concomitantly with immunotherapy in local/locoregionally or in oligometastatic disease, different from this case. SBRT was performed, with a total dose of 50 Gy to the upper right lobe divided into five fractions, which is a commonly used dose for SBRT in early-stage NSCLC with curative intent. The major concern of the treatment association was toxicity, especially pneumonitis that was not observed. Our patient had common and easily manageable toxicities such as endocrinopathies and gastrointestinal enterocolitis, and neither were severe nor needed immunotherapy suspension, nor hospital admission.

Finally, it is important to discuss the best treatment in case malignant disease returns. In the KEYNOTE-042 trial, patients who completed the 35 cycles of pembrolizumab or who stopped pembrolizumab after achieving a complete response and had disease progression were eligible for a second course of monotherapy pembrolizumab. This second course was feasible and associated with antitumor activity [16]. Other studies have addressed this issue, showing evidence that restarting immunotherapy in patients who initially benefited from it is considered safe and effective [17]. Despite this, more studies are required to clarify the benefit of immunotherapy rechallenge. Therefore, regarding the previous good response and tolerance to pembrolizumab, a second course would be a treatment option for our patient if he had disease progression.

## Conclusions

Monotherapy immunotherapy in patients with advanced NSCLC that express high levels of PD-L1 has already shown efficacy and durable responses, which are not usually seen with other systemic treatments in clinical practice, with a good safety profile. Our case represents an example of success, demonstrating a great tumor response with immunotherapy that allowed a patient with advanced non-metastatic NSCLC whose disease had progressed with platinum-based chemotherapy to get radical treatment with SBRT. The failure of the first-line treatment can appoint more investigation on the efficacy and benefits of beginning treatment of these kinds of tumors with ICI directly.
